# Results of the 2014–2015 Canadian Society of Nephrology workforce survey

**DOI:** 10.1186/s40697-016-0117-6

**Published:** 2016-05-12

**Authors:** David R. Ward, Braden Manns, Sarah Gil, Flora Au, Joanne E. Kappel

**Affiliations:** Department of Medicine, University of Calgary, Calgary, Alberta Canada; Division of Nephrology, Department of Medicine, University of Saskatchewan, Rm 434- 230 Avenue R South, Saskatoon, SK S7M 2Z1, Saskatchewan Canada

**Keywords:** Workforce, Nephrology, Human resource, Employment, Health manpower

## Abstract

**Background:**

Nephrology was previously identified as a subspecialty with few Canadian employment opportunities, and in recent years, fewer trainees are choosing nephrology.

**Objective:**

The objective of this study is to better understand the current Canadian adult nephrology workforce and the expected workforce trends over the next 5 years.

**Design:**

This is an online self-administered survey.

**Setting:**

This study is set in Canada.

**Survey participants:**

Survey participants are Canadian adult nephrologists, including self-identified division heads.

**Measurements:**

The measurements of this study are demographics, training, current practice characteristics, work hours, and projected workforce needs.

**Methods:**

Survey questions were based on previous workforce surveys. Ethics approval was obtained through the University of Saskatchewan. The survey was piloted in both English and French and modified based on the feedback to ensure that responses accurately reflected the information desired. It was circulated to all identified Canadian nephrologists via an anonymous e-mail link for self-administration. Categorical data was aggregated, and free-text answers were thematically analyzed. Additional descriptive analysis was conducted by all authors.

**Results:**

Five hundred ninety-two Canadian nephrologists were contacted and 48 % responded, with representation from all Canadian provinces. One third of the respondents were female, and the largest age cohort was 41–50 years. Most nephrologists are trained in Canada and 61 % completed additional training. The majority of the respondents (69.1 %) began working as a nephrologist immediately upon completion of fellowship training. Younger nephrologists reported more challenges in finding a job. Eighty percent of responding nephrologists were satisfied with their current work hours, 13.1 % will reduce work hours within 3 years, an additional 8.2 % will reduce work hours within 5 years, and a further 14.2 % will reduce work hours within 10 years. Nephrology division heads forecasted the number of clinical and academic nephrologists needed for the next 3 and 5 years.

**Limitations:**

The response rate was 48 %. Forecasted workforce needs are not indicative of guaranteed future positions.

**Conclusions:**

This Canadian Society of Nephrology workforce survey demonstrated the current workforce demographics, individual nephrologist future workforce plans, and projected nephrology division requirements for the next 3 and 5 years. Further work will need to be done to refine Canadian nephrology workforce planning with the development of a robust strategy that encompasses both societal and nephrologists’ needs with the realities of employment.

**Electronic supplementary material:**

The online version of this article (doi:10.1186/s40697-016-0117-6) contains supplementary material, which is available to authorized users.

## Abrégé

**Mise en contexte:**

La néphrologie a été identifiée dans le passé comme une sous-spécialité pour laquelle les perspectives d’emploi se faisaient rares au Canada. Une conséquence de cette constatation a été la diminution du nombre de stagiaires qui l’ont choisie comme spécialité dans les dernières années.

**Objectifs du sondage:**

On a cherché à mieux connaître la nature des effectifs actuels en néphrologie adulte au Canada et à mesurer l’évolution de la main-d’œuvre pour les cinq prochaines années.

**Type d’étude:**

Le plan de l’enquête reposait sur un questionnaire autoadministré auquel les participants accédaient en ligne.

**Participants:**

Le sondage s’est tenu auprès des néphrologues canadiens traitant des adultes et incluait des répondants s’identifiant comme chefs de division.

**Mesures:**

On a recueilli des données sur les effectifs actuels la formation, les caractéristiques des pratiques en vigueur, le nombre d’heures travaillées et les besoins anticipés à moyen terme en fait de main d’œuvre.

**Méthodologie:**

Les questions de la présente enquête ont été établies à partir de sondages réalisés antérieurement au sujet des effectifs. L’Université de la Saskatchewan a fourni les approbations déontologiques nécessaires à la conduite de l’enquête. Le questionnaire modifié sur la base des commentaires reçus dans les sondages précédents pour s’assurer que les réponses tenaient compte de l’information souhaitée, était disponible en anglais et en français. Le questionnaire a été distribué à tous les néphrologues canadiens par le biais d’un lien de courrier électronique anonyme afin que ceux-ci puissent le remplir de façon autonome. Les données catégorielles ont été comptabilisées et les réponses aux questions ouvertes ont fait l’objet d’une analyse thématique. Enfin, les auteurs du présent article ont procédé à des analyses descriptives supplémentaires.

**Résultats:**

Le questionnaire a été envoyé à 592 néphrologues ceux-ci ont répondu dans une proportion de 48 % et celle-ci comptait des représentants de toutes les provinces canadiennes. Le tiers des répondants étaient des femmes, et le groupe d’âge prédominant était celui des 41 à 50 ans. La plupart des néphrologues questionnés avaient reçu leur formation au Canada, une proportion de 61 % avait suivi une formation supplémentaire. La majorité des répondants (69,1 %) ont mentionné avoir trouvé un emploi dans leur domaine de spécialité dès la fin de leur formation postdoctorale, mais les plus jeunes ont rapporté avoir rencontré plus de difficultés au moment de leur recherche d’un poste de néphrologue. La grande majorité des répondants (80 %) se disait satisfaite du nombre d’heures travaillées actuellement. Cependant, 13,1 % des néphrologues en poste ont l’intention de réduire leurs heures de travail au cours des trois prochaines années, 8,2 % prévoient de le faire dans les cinq ans alors que 14,2 % envisagent de le faire d’ici dix ans. Quant aux besoins d’effectifs en néphrologie clinique ainsi que pour le volet académique, les chefs de division ont fait des prévisions pour les prochains trois et cinq ans.

**Limites de l’étude:**

Le taux de participation au sondage ne s’élevait qu’à 48 %. De plus les prévisions concernant les besoins de main-d’œuvre mentionnés dans cette publication ne reflètent pas nécessairement de futures affectations.

**Conclusions:**

Cette enquête sur la situation des effectifs menée par la Société Canadienne de Néphrologie dresse le portrait de la situation actuelle des plans d’avenir individuels des néphrologues, de même que des besoins d’effectifs anticipés par les chefs de division pour les prochains trois et cinq ans. Des travaux ultérieurs devront être réalisés pour mieux arrimer la planification de la main-d’œuvre en néphrologie au Canada au développement de stratégies solides se penchant à la fois sur les besoins sociétaux et ceux des néphrologues concernant les réalités du marché du travail.

## What was known before

The last workforce survey of Canadian nephrologists was in 2007. Interest in nephrology as a career in Canada appeared to be declining, and nephrology was identified as a Canadian specialist area with few employment opportunities. The decline in interest in nephrology in other jurisdictions has been linked to perceptions of poor job satisfaction.

## What this adds

We present data on the most current demographics, training and employment search experiences, workload, job satisfaction, and career plans of the Canadian nephrology workforce as well as information obtained from nephrology division heads on the perceived needs of Canadian nephrology divisions over the next 5 years.

## Background

In 2010, the Royal College of Physicians and Surgeons of Canada (RCPSC) reported that there were few employment opportunities within Canadian nephrology [1]. A subsequent RCPSC study found that the rate of newly certified, sub-specialist physicians without a job placement increased from 15 to 21 % between 2011 and 2012 [1]. More recent statistics from the Canadian Resident Matching Service suggest fewer Canadian internal medicine trainees chose nephrology. In 2014, 4.5 % of candidates chose nephrology as their first choice, a decline from 6.2 % in 2010 [2, 3]. This trend is not unique to Canada; recent data from the United States of America (US) also suggests a declining interest in nephrology as a career [4]. Many reasons for the declining interest in a career as a nephrologist have been suggested, including concerns about job availability and work-life balance [4–10].

The Canadian Society of Nephrology (CSN) previously surveyed identified leaders of nephrology divisions and practice groups in 2007 (personal communication, Barrett B). Respondents perceived there would be increased workload due to a rise in patient numbers with end-stage renal disease (ESRD) and chronic kidney disease (CKD). Although few nephrologists were believed likely to retire within the subsequent 5 years, an additional 60 nephrologists encompassing 38 full-time equivalent (FTE) nephrology positions nationwide were felt to be required in the same time frame to address growing demand.

Physician workforce planning is complex. Health-care resources and societal needs must be balanced, and factors such as changing models of care, a weaker Canadian economy, delayed retirements, and reduced health-care expenditures play a major role in physician training, employment, and future workforce planning. Given the increasing numbers of people with CKD, increasing prevalent rates of patients receiving dialysis and/or renal transplant, more reliance on team-based care, and the perception that there were very few career opportunities in nephrology, the CSN launched a survey of Canadian nephrologists to better understand the demographics and future plans of the current workforce, to determine the hiring needs of nephrology divisions across Canada over the next 5 years and to serve as a foundation for more in-depth strategic workforce planning activities.

## Methods

The survey questions were developed from reviews of nephrology workforce reports from Australia and New Zealand, the United States, and the United Kingdom as well as input from Canadian nephrology leaders [8, 9, 11, 12].

A preliminary draft of the survey was sent to a convenience sample of Canadian nephrology trainees and junior and senior adult nephrologists including division heads, full-time clinicians, and clinician-scientists. They were asked to complete the survey and make suggestions for possible changes, additions, or deletions. Survey themes included demographics, training and current practice characteristics, work hours, and additional health resource utilization and optimization. Further, an additional embedded survey of nephrology division heads asked about the current number of clinical (defined as ≥75 % of workload time spent on direct patient care-related activities) and academic (defined as ≥75 % of workload time spent teaching/research-related activities) FTE (defined as working 44 h per week for 44 weeks per year (approximately 2200 h/year), excluding on-call duties) nephrologists as well as projected clinical and academic human resource requirements for the next 3 and 5 years.

The final survey, available in both French and English (see Additional file 1; CSN 2014–2015 Workforce Survey Questions), was hosted by FluidSurveys™ who collected and stored the primary data. All respondents gave consent prior to completing the survey questions. Unique survey links were sent to e-mail addresses of all known nephrologists in Canada identified through the CSN’s protected e-mail list or through public records and comprehensive provincial lists for nephrologists who were not CSN members. The number of contacts was compared to available statistics from the Canadian Institute for Health Information (CIHI) as a measure of completeness because no official contact list of actively practicing nephrologists in Canada is available. Division heads were asked to self-identify.

Respondents included those who had completed nephrology training. Individuals who had retired could not complete the survey. Respondents did not have to answer all questions to be included in the survey results. To promote completion of the survey, respondents had the opportunity to enter their name into a random draw for a prize after completing the survey. The first e-mail containing the link was sent in November 2014, with two follow-up reminders sent every 4 weeks in December 2014 and January 2015. Prior to the closing of the survey in February 2015, D.W. contacted all known nephrology division heads who had not responded to the survey to verify receipt of the survey and to encourage participation. In late 2015, prior to publication, nephrology division heads were again contacted to confirm the accuracy of previously provided recruitment data.

Prior to analysis, all data reported from the final survey was de-identified and aggregated. Only the authors and data analysts had access to the raw responses. For textual responses, primary thematic analysis was carried out by J.K. and confirmed by D.W. Disagreement was resolved through consensus.

### Ethics approval

Ethics approval was obtained from the University of Saskatchewan Behavioral Research Ethics Board.

## Results

A total of 592 unique e-mail addresses affiliated with known adult Canadian nephrologists were identified. The number of nephrologists registered with the CSN and the number on the CIHI database were comparable. After closure of the survey, three individuals were removed from the analysis because they opened the e-mail link and viewed the survey but did not answer any questions. The average time to complete the survey was 15 min. Two hundred eighty-two responses (48 % response rate) were received and analyzed, including 50 who identified themselves as a nephrology division head or responsible for their local nephrology recruitment. These division heads were from both academic institutions and community practices. Some responders did not answer every question. At least one response from a nephrologist and division head was received from each Canadian province.

### Demographics

One third (33.3 %) of the overall respondents were female. The largest age cohort was 41–50 years old (range 30 to >70 years old) and 12.1 % of the respondents were aged 61 or older (Table 1). The majority (96.5 %) of nephrologists stated they only practiced in one province, and 192 respondents (68.1 %) stated they practiced in an urban center with a catchment area of greater than 500,000 people. Most Canadian nephrologists obtained their subspecialty training within Canada with only 11.4 % of respondents stating their nephrology training was obtained outside of Canada, most commonly in the United States or Europe.

**Table 1:** Demographics (*n* = 282)

Female, *N* (%)	94 (33.3)
On leave, *N* (%)	4 (1.4)
Age, *N* (%)	
31–40	70 (24.8)
41–50	112 (39.7)
51–60	65 (23.1)
61–70	27 (9.6)
>70	7 (2.5)
No answer	1 (0.4)
Primary province of practice, *N* (%)	
British Columbia	41 (14.5)
Alberta	37 (13.1)
Saskatchewan	11 (3.9)
Manitoba	18 (6.4)
Ontario	97 (34.4)
Quebec	57 (20.2)
New Brunswick	7 (2.5)
Nova Scotia	11 (3.9)
Newfoundland and Labrador	2 (0.7)
Prince Edward Island	1 (0.4)
Catchment population size, *N* (%)	
<100,000	5 (1.8)
100,001–250,000	35 (12.4)
250,001–500,000	50 (17.7)
500,001–1,000,000	67 (23.8)
>1,000,001	125 (44.3)

### Training

Additional training beyond RCPSC or equivalent basic nephrology certification was undertaken by 172 (61.0 %) of the respondents, and the most common additional training was a master’s degree (*N* = 76), followed by a clinical fellowship in fields such as dialysis or glomerulonephritis (*N* = 62), PhD and post-doctoral work (*N* = 60), and 33 other responses encompassing areas such as health leadership/administration, palliative care, and education. A minority (*N* = 7, 4.1 %) stated they obtained this training before undertaking their nephrology training. Most individuals (*N* = 114, 66.3 %) completed this training to improve their chances of finding employment at their own discretion or the advice/direction of colleagues. A large proportion (*N* = 73, 42.4 %) undertook additional training not for employment reasons but for personal passion or growth. Of those that had completed additional training, 144 individuals (83.2 %) utilized their training in their routine practice.

### Employment search experience

The majority of respondents (*N* = 195, 69.1 %) were able to begin working as a nephrologist immediately after completing their nephrology training. A minority (*N* = 10, 3.3 %) required greater than 5 years to become employed, and four respondents (1.4 %) stated they were still not working as a Canadian nephrologist at the time of the survey. We also asked respondents to rank their top three most helpful resources for finding a job. Of the 275 individual rankings received, the options most commonly cited were word of mouth (27 %), colleagues (22 %), division heads (18 %), and program directors (13 %). Other options such as CSN meeting(s), CSN website, and social media were selected much less frequently.

Additionally, 23 individuals provided written responses that revealed additional strategies and processes for obtaining employment including the following:

- Individual-initiated contact with division heads: “I wrote to heads of division(s) in places where I thought I would like to live”
- Service and other rotations: “I did electives [during training] and locums…”
- Leveraging of relationships with other physicians: “[a] colleague in another specialty who knew of an opening…”
- And in one jurisdiction, respondents noted that the government mandates their location of employment based on the population characteristics and their training.

One third of respondents (*N* = 91, 31.2 %) stated they experienced at least one challenge in finding employment. A total of 156 challenges were reported, and the three most common were an inability to find a job in their location of choice, inconsistent advertising practices across jurisdictions, and that openings were perceived to be filled even before jobs were advertised (Table 2). Younger respondents aged less than 50 years old were more likely to state they had experienced at least one challenge in finding a job. Written responses were received from 22 individuals, and many described negative experiences and were critical of the processes in place during their employment search.

**Table 2:** Challenges experienced in getting a job as a nephrologist by age cohort N (% of age cohort)

	Overall^a^ (*n* = 282)	Age 31–40^a^ (*n* = 70)	Age 41–50^a^ (*n* = 112)	Age 51–60^a^ (*n* = 65)	Age 61–70^a^ (*n* = 27)	Age >70^a^ (*n* = 7)
No challenge finding a job in nephrology	191 (67.7)^b^	38 (54.3)	75 (67.0)	51 (78.5)	21 (77.8)	5 (71.4)
I could not find a nephrology job in Canada	5 (1.8)	2 (2.9)	0 (0.0)	1 (1.5)	1 (3.7)	1 (14.3)
I could not find a nephrology job in the location of my choice	37 (13.1)	16 (22.9)	14 (12.5)	6 (9.2)	1 (3.7)	0 (0.0)
I could not find a nephrology job where my particular skills could be used	10 (3.5)	6 (8.6)	4 (3.6)	0 (0.0)	0 (0.0)	0 (0.0)
Non-nephrology job-related factors, e.g., no job available for my spouse or significant other	10 (3.5)	4 (5.7)	4 (3.6)	2 (3.1)	0 (0.0)	0 (0.0)
Job vacancies appeared to be filled/candidate selection made prior to the advertisement posting	29 (10.3)	14 (20.0)	11 (9.8)	3 (4.6)	1 (3.7)	0 (0.0)
Canadian immigration, language, or training restrictions/requirement	8 (2.8)	1 (1.4)	5 (4.5)	0 (0.0)	2 (7.4)	0 (0.0)
Difficulty identifying job vacancies in Canada due to inconsistent posting	34 (12.1)	14 (20.0)	15 (13.4)	2 (3.1)	2 (7.4)	1 (14.3)
Others	23 (8.2)	7 (10.0)	8 (7.1)	5 (7.7)	2 (7.4)	1 (14.3)

^a^Adds to >100 % as respondents could select more than one answer

^b^One survey respondent did not state their age

- “It is challenging to find a full time nephrology job… and a lot of it is done by word of mouth and soliciting programs. Few divisions, if any, advance plan and advertise well externally”
- “…the process was opaque with[out] a clear process or guarantees”
- “Difficulty knowing what the job would entail”
- “Anyone coming from the outside is not viewed favorably”

### 2014–2015 Canadian nephrology practice characteristics

**Fig. 1:**
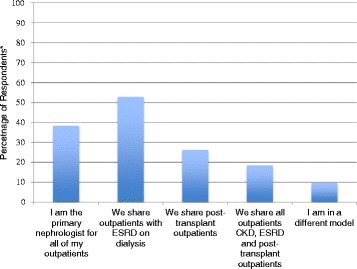
Most common patient care models identified by Canadian nephrologists

Although the vast majority of Canadian adult nephrologists (*N* = 243, 86.2 %) defined their practice as adult nephrology only, five individuals (1.8 %) also practiced pediatric nephrology and thirty-four also practiced another subspecialty, most commonly, general internal medicine and/or intensive care. The majority (89.0 %) also stated they practiced in a group as opposed to solo environment and were (at least partially) affiliated with an academic center (e.g., teaching nephrology fellows in a community center). The structure of caring for patients differed widely (Fig. 1) and the number of patients cared for by an individual nephrologist, stratified by CKD stage, varied (Table 3). When asked to rank their satisfaction with their overall position on a scale from 1 (least satisfied) to 10 (most satisfied), the average score was 8 ± 1.7. However, 10 % of respondents rated their satisfaction as 5 or less.

**Table 3:** Self-reported outpatient practice size by CKD status

	Min.	25 % Percentile	Median	75 % Percentile	Max.^a^
CKD stages 1–3	0	100	200	300	2000
Non-dialysis CKD stages 3–5	0	100	185	300	2000
ESRD on peritoneal dialysis	0	0	10	25	185
ESRD on hemodialysis	0	23	47.5	77.5	400
Post-transplant	0	0	0	50	1600

^a^All reported maximum values were provided by nephrologists who share the patients in their practice

Just over one-half of respondents (*N* = 149, 52.8 %) stated their remuneration model was fee-for-service. Of the remainder, most received some or all of their income from alternative relationship/funding plans (*N* = 107) while a very small number of individuals were compensated with a salary. Additional textual responses from 21 respondents revealed other strategies for compensation, and the most commonly quoted was a model where all group members pool income and distribute based on seniority.

### 2014–2015 Canadian nephrology workload characteristics

Nephrologists worked, on average, 51 ± 16 h (range 5 to >100 h) per week as a nephrologist (excluding on call). The number of daytime hours per week had, for the majority, not changed significantly in the past 2 years. Nephrologists were on-call an average of 62 ± 48 nights per year including weekends (range 0 to 365 nights) and, for the majority, the number of nights on-call had also not changed significantly in the past 2 years. To assist in the management of the workload related to the care of patients, many nephrologists (*N* = 193, 68 %) acknowledged the use of additional, advanced care providers such as nurse practitioners (*N* = 132), pharmacists (*N* = 76), or physician assistants (*N* = 36) in their daily work. When asked to rank their satisfaction with their work hours on a scale from 1 (least satisfied) to 10 (most satisfied), the average score was 7 ± 2. However, 20 % of respondents (*N* = 56) rated their satisfaction as 5 or less.

**Fig. 2:**
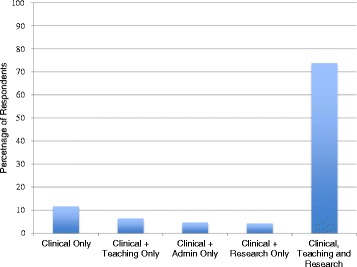
Reported composition of annual workload of Canadian nephrologists

Most nephrologists (*N* = 275) were able to describe the composition of their annual responsibilities according to clinical work, teaching, administrative duties, research, and “other” activities such as continuing education. Only a minority of respondents reported they were clinicians only, and the remainder undertakes varied responsibilities as teachers, administrators, and educators (Fig. 2).

### Divisional composition

Canadian nephrology division heads (defined as individuals who would be responsible for hiring additional nephrologists if resources permitted) revealed that, on average, Canadian nephrology divisions contained 6.9 FTE clinical nephrologists. The number of FTE clinical nephrologists varied significantly between divisions, from 0 to 19. Division heads reported an average of 6.8 FTE and a range of 0 to 18 FTE academic nephrologists per division. Of the individual nephrologists hired within each division, 72 % of clinical nephrologists were working 1.0 FTE and 28 % of academic nephrologists were working 1.0 FTE.

### Individual future workforce plans

Almost one-half (*N* = 129, 46 %) of respondents stated they will reduce their working hours by some proportion in the next 15 years. Thirty-seven respondents (13.1 %) believed they would reduce their daytime hours by a median of 33 % (range 10 to 100 %) within 3 years, with an additional 23 (8.2 %) reducing hours by a median of 25 % (range 10 to 50 %) within 5 years, a further 40 (14.2 %) reducing their working hours by a median of 28 % (range 0 to 100 %) within 10 years, and 29 (10.3 %) reducing their working hours by a median of 30 % (range 10 to 100 %) within 15 years. The remainder (*N* = 153, 54 %) stated that within the next 15 years, they were either not planning to or were unsure if they would reduce their daytime working hours.

A total of 13 nephrologists stated they would retire within the next 3 years, a further 21 within 5 years, 28 additional nephrologists would leave the workforce within 10 years, and 44 more would retire within 15 years; a total of 106 planned retirements over the next 15 years.

### Divisional future workforce plans

**Fig. 3:**
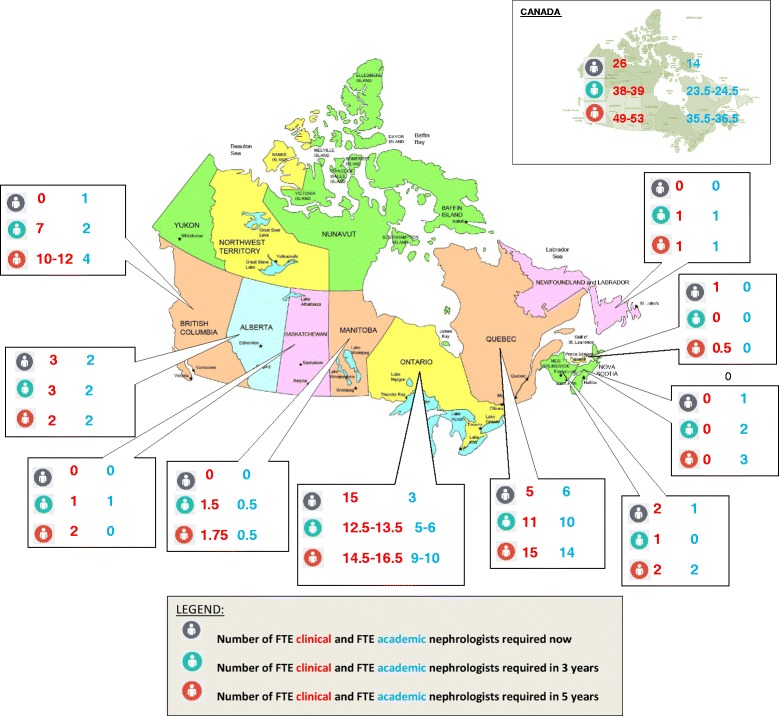
Future FTE workforce needs (if all required resources were available)

Nephrology division heads were asked about future FTE workforce needs (if all required resources were available)—now, in 3 years (excluding immediate hires) and in 5 years (excluding immediate and 3-year hires). Forty-four of the 50 original respondents re-confirmed their forecast for human resource needs prior to publication. For those who did not re-confirm, the original values provided were used (Fig. 3). In summary, Canada-wide, 26 FTE clinical nephrologists (defined as ≥75 % of workload time spent on direct patient care-related activities) are needed immediately, with an additional 38–39 FTEs in the next 3 years and a further 49–53 FTEs in 5 years for a total of 113–118 FTE clinical nephrologists in the next 5 years. Furthermore, 14 FTE academic nephrologists (defined as ≥75 % of workload time spent teaching/research-related activities) are desired immediately, an additional 23.5–24.5 FTE academics within 3 years and 35.5–36.5 additional FTE academic nephrologists in 5 years with a total of 73–75 FTE academic nephrologists over the next 5 years.

## Discussion

Physician workforce planning is complex [13–16]. Many factors including patient population characteristics, funding and policy structures, trainee numbers, an individual’s expertise, an individuals’ priorities and aspirations, the local economy, and the current workforce’s forecasted changes influence the outcome [1, 17–20]. Given concerns raised by trainees and the RCPSC, and to inform workforce planning, the CSN undertook a voluntary, comprehensive workforce survey of all nephrologists in Canada. Although our response rate of 48 % is lower than that achieved in other physician surveys, it confirms that collecting data on the current and projected Canadian nephrology workforce is possible [11, 21, 22]. We were able to survey a significant number of division heads. Future surveys conducted by the CSN at regular intervals are expected to have higher response rates as the relevance of the survey to division heads, trainees, and others involved in workforce planning improves. Furthermore, our challenges in contacting nephrologists and collecting this important data will hopefully lead to a national, well-maintained database of trained nephrologists within Canada. Future surveys would then allow all who would want to respond, including those who completed training but are not working as nephrologists, the opportunity to do so.

**Fig. 4:**
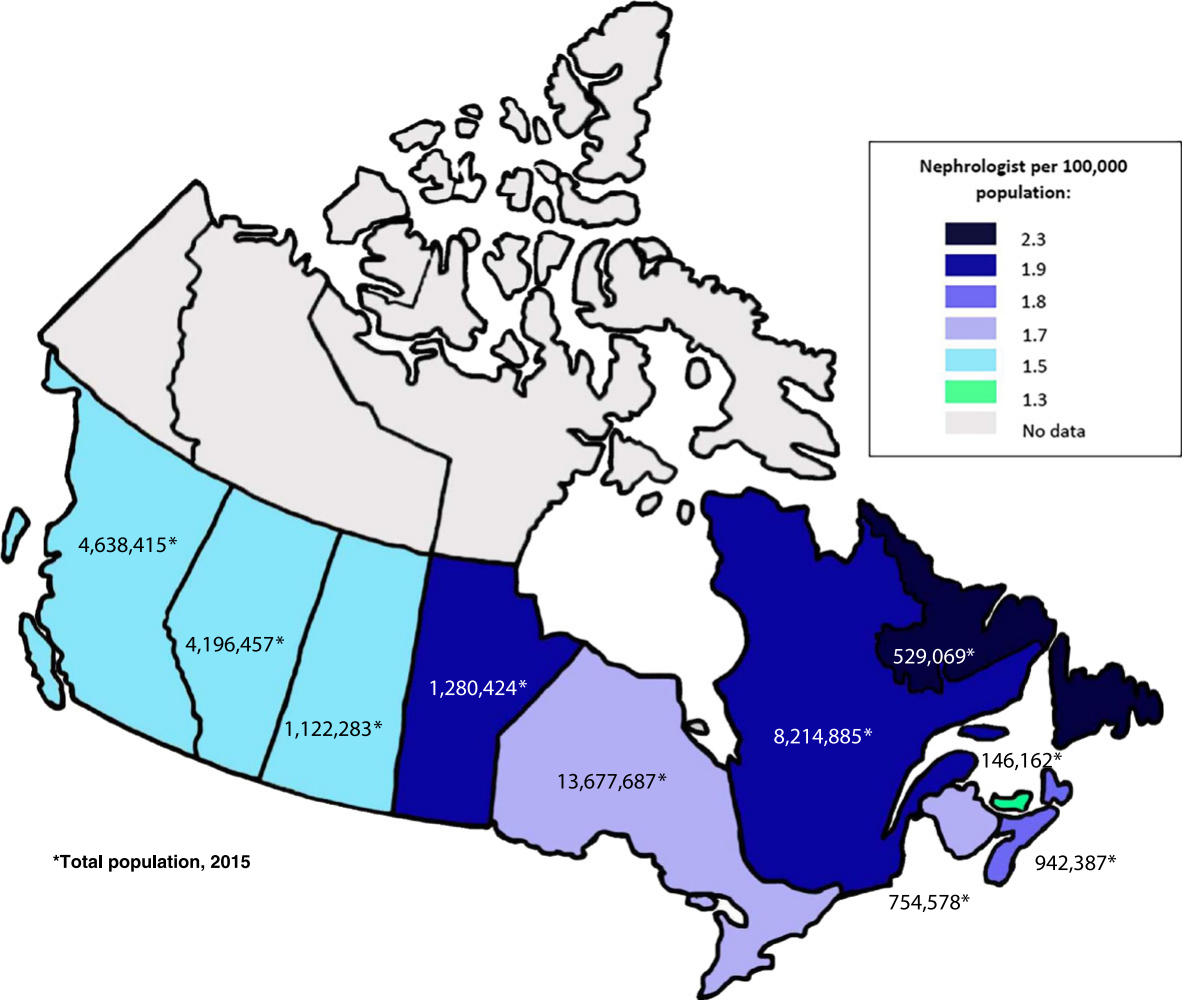
The average number of nephrologist per hundred thousand population

We found that the Canadian nephrology workforce as of 2014–2015 was relatively young, with two thirds of respondents under 50 years. The majority also worked in group practices and in areas with a catchment above 500,000 population. Based on our compiled contact list, we also determined that the average number of nephrologists per hundred thousand population was 1.4–2.3 depending on the province (Fig. 4). In contrast, recent reports from the US revealed an average of 2.7 and up to 6.3 adult nephrologists per hundred thousand population in some states [8, 23]. Future studies should explore variation in the number of patients with CKD, including end-stage renal disease per 100,000 population, to the number of nephrologists serving them for additional insights into the workload facing nephrologists, nephrology divisions, and the overall health-care system.

A key development that led to the launch of this survey was reports of job-finding difficulties amongst newly trained nephrologists [1, 8, 9]. Although 31.2 % of our respondents experienced employment challenges, the reasons cited were multifactorial and related to both the individual (an inability to find a job in the location of their choice) and the systemic processes used by divisions for hiring (e.g., inconsistent advertising); findings similar to those in the US [9]. In contrast to this same report from the US, we learned that the vast majority of Canadian nephrologists have completed their basic nephrology training in Canada. Furthermore, 61 % went on to obtain additional training, most often a masters or clinical fellowship. Although many pursued training to enhance a personal passion, additional training was most often undertaken to increase employability—a finding consistent with the RCPSC 2013 report. It is worth noting that respondents reporting challenges in job seeking were more commonly younger, presumably more recently trained, nephrologists. Furthermore, a proportion of those who experienced challenges stated that there were no jobs where their particular skills, presumably gained through extra training, could be used. This may suggest there is opportunity for clearer communication between trainees and divisions regarding training after certification. Reassuringly, regardless of the reason, Canadian nephrologists stated that their extra training was utilized in their routine daily practice. A future project linking the number of Canadian trainees per year to the expected/desired recruitment needs in 3–5 years including the upcoming change to nephrology training (competency-based education) is planned and should be completed by the end of 2016.

Additional concerns purported to have reduced the numbers of nephrology trainees are those related to work-life balance. We found that Canadian nephrologists work 51 h/week, a number almost identical to similar studies in Australia and the US [11, 24]. Canadian nephrologists also spend on average 60 nights on-call per year. Average job satisfaction amongst our responders was rated as 7/10 for work hours and 8/10 for overall career status, but it is worth noting that between 10 and 20 % rated their satisfaction as <5/10. These findings are similar to a study looking at job satisfaction in the US [5]. Future studies should explore if temporal relationships exist between employment challenges/career stage and work-life balance. It may be that more challenges and lower satisfaction scores with hours of work and overall position are more common in junior staff members and recent graduates.

We found that 34 nephrologists are planning to completely retire within the next 5 years and a further 28 will retire within the subsequent 5 years. Furthermore, 46 % of nephrologists are planning to reduce their work hours by some proportion within the next 15 years. However, caution should be given to interpreting these results as internal or external factors to an individual nephrologist such as personal health or economic market performance may either accelerate or delay retirements.

Given these predicted workforce changes and the estimated increases in CKD and ESRD, the projected need for 113–118 FTE clinical nephrologists and 73–75 academic nephrologists over the next 5 years in Canada is likely accurate. Recent data from the Canadian Resident Matching Service [2, 3, 25], and preliminary data from our upcoming report, suggest that approximately 20–30 residents (0–3 residents per program) enter into Canadian nephrology training programs each year, and supply may therefore not meet demand. Furthermore, numerous factors beyond the number of persons graduating will influence whether or not those entering the workforce will effectively replace those exiting, including plans for extended training (and thus delayed employment), workload composition (clinical vs. research, for example), and work-life balance; this deserves further study. Finally, it deserves mention that these projected requirements are based on anticipated needs and desires of nephrology divisions and given the complex nature of hiring physicians should not be interpreted to represent guaranteed future positions.

## Conclusions

In summary, this survey is an important contribution to understanding the Canadian nephrology workforce and developing a strategy to ensure a sufficient future nephrology workforce in Canada. It has revealed features of the current workforce including real-world employment challenges, what the future plans of nephrologists are, and what the projected needs of Canadian nephrology divisions are and could be in the future. Further work needs to be done to develop a comprehensive Canadian nephrology workforce strategy.

### Availability of data and materials

To protect the identity of respondents, the dataset supporting the conclusions of this article are not publically available. The survey questions are attached as an Additional file 1.

## Additional file

Additional file 1: CSN 2014–2015 Workforce Survey Questions. The complete set of the survey questions administered to Canada’s nephrologists. (PDF 100 kb)

## Abbreviations

CIHI: Canadian Institute for Health Information
CKD: chronic kidney disease
CSN: Canadian Society of Nephrology
ESRD: end-stage renal disease
FTE: full-time equivalent
RCPSC: Royal College of Physicians and Surgeons of Canada
US: United States of America
