# Supplementary material for: Results of the 2014–2015 Canadian Society of Nephrology workforce survey
Source: Can J Kidney Health Dis. 2016 May 12;3:25. doi: 10.1186/s40697-016-0117-6 (PMC4864912; doi:10.1186/s40697-016-0117-6)
Supplement: Additional file 1: — CSN 2014–2015 Workforce Survey Questions. The complete set of the survey questions administered to Canada’s nephrologists. (PDF 100 kb) [file 40697_2016_117_MOESM1_ESM.pdf]

CSN-Physician Workforce Survey 2014

Canadian Society of Nephrology/
Société canadienne de néphrologie
CSN/SCN

CSN Physician Workforce Survey 2014:

Researcher(s):
Joanne Kappel, B.Sc., M.D., FRCPC
Chair, Canadian Society of Nephrology Workforce Planning Survey Committee
Clinical Professor of Medicine
Head, Division of Nephrology
Dyad Co-Lead, Renal Services
University of Saskatchewan
Saskatoon Health Region
Rm 434-230 Avenue R South
Saskatoon, SK S7M 2Z1
Phone: 306-934-3300
Fax: 309-934-3355
Email: jok762@mail.usask.ca

This survey is designed to help us understand: The current demographics and workload of Canadian Nephrologists The future career plans of Canadian Nephrologists, and The recruitment plans of Canadian Nephrology divisions including types of recruitment projected.
This information will help to identify and understand this complex and multifaceted aspect of the Canadian health care system and will help in developing a future workforce strategy for Canadian Adult and Pediatric Nephrology. This information will also help inform Nephrology Training Programs, and present/future Academic and Clinical nephrology requirements.

Participation in this survey is voluntary and you can decide not to participate at any time by closing your browser or choose not to answer any questions you do not feel comfortable with. Survey responses will remain anonymous. Since the survey is anonymous, once it is submitted it cannot be removed.

There are no known risks to participating in this survey.

This survey is hosted by Fluid Survey, a USA owned company and subject to US laws and whose servers are located outside of Canada. The privacy of the information you provide is subject to the laws of those other jurisdictions. By participating in this survey you acknowledge and agree that your answers/information will be stored and accessed outside of Canada and may or may not receive the same level of privacy protection.

Completion of the survey should take 10-20 minutes depending on your status as a nephrologist.

This research project has been approved on ethical grounds by the University of Saskatchewan Research Ethics Board Any questions regarding your rights as a participant may be addressed to that committee through the Research Ethics Office ethics.office@usaks.ca (306) 966-2975. Out of town participants may call toll free (888) 966-2975.

By completing and submitting this questionnaire, your free and informed consent is implied and indicates that you understand the above conditions of participating in this study.

After completing this survey you will have the opportunity to enter your email address for a chance to win an Apple Ipad. If you choose to enter your email address it will not be linked to any of your prior responses.

Have you completed training in nephrology (even if you are not currently working as a nephrologist)?

- ◯ Yes
- ◯ No

Simple Skipping Information

- If 5. Do you travel to another province to provide Ne... = Yes then Skip to Page 3
- If 5. Do you travel to another province to provide Ne... = No then Skip to Page 4

DEMOGRAPHICS:

1. Are you:

- Male
- Female

2. What is your age:

- 20-30
- 31-40
- 41-50
- 51-60
- 61-70
- >70

3. After you finished your clinical Nephrology training, how many years did it take you to find employment as a Canadian Nephrologist?

- 0 years (I started right away)
- 1 year
- 2 years
- 3 years
- 4 years
- 5 years
- 5-10 years
- >10 years
- I'm still not employed as a Canadian Nephrologist

4. What is your primary province of practice:

- British Columbia
- Alberta
- Saskatchewan
- Manitoba
- Ontario
- Quebec
- New Brunswick
- Nova Scotia
- Newfoundland
- Prince Edward Island
- Northwest Territories
- Nunavut
- Yukon

5. Do you travel to another province to provide Nephrology services?

- Yes
- No

5 a. Please check all additional provinces you travel to as a Nephrologist.

- [ ] British Columbia
- [ ] Alberta
- [ ] Saskatchewan
- [ ] Manitoba
- [ ] Ontario
- [ ] Quebec
- [ ] New Brunswick
- [ ] Nova Scotia
- [ ] Newfoundland
- [ ] Prince Edward Island
- [ ] Northwest Territories
- [ ] Nunavut
- [ ] Yukon

6. Geographic catchment of primary practice:

- <100,000
- 100,000 - 250,000
- 250,000 - 500,000
- 500,000 - 1, 000,000
- >1,000,000

7. Are you a member of the Canadian Society of Nephrology (CSN)?

- Yes
- No

Simple Skipping Information
- If 2. Are you retired? = Yes then Skip to Page 30
- If 2. Are you retired? = No then Skip to Page 6

TRAINING AND CURRENT PRACTICE CHARACTERISTICS:

1. Where did you complete your Nephrology fellowship training?

- University of British Columbia, Vancouver BC
- University of Calgary, Calgary AB
- University of Alberta, Edmonton AB
- University of Saskatchewan, Saskatoon SK
- University of Manitoba, Winnipeg MB
- Western University, London ON
- McMaster University, Hamilton ON
- University of Toronto, Toronto ON
- Queen’s University, Kingston ON
- University of Ottawa, Ottawa ON
- McGill University, Montreal QC
- Université de Montréal, Montréal QC
- Université de Sherbrooke, Sherbrooke QC
- Université Laval, Québec, QC
- Dalhousie University, Halifax NS
- Memorial University of Newfoundland, St. John’s NL
- Outside Canada (please specify) ________________________

2. Are you retired?

- Yes
- No

Simple Skipping Information

- If 3. Are you temporarily on leave? = Yes then Skip to Page 7
- If 3. Are you temporarily on leave? = No then Skip to Page 8

3. Are you temporarily on leave?

- Yes
- No

Although you have indicated you are on leave, please answer the following questions for when you return to work.

Simple Skipping Information

- If 4.Have you completed any specific training/degree(... = Yes then Skip to Page 9
- If 4.Have you completed any specific training/degree(... = No then Skip to Page 10

4.Have you completed any specific training/degree(s), in addition to the Royal College of Physicians and Surgeons of Canada minimum requirements to practice as a Nephrologist (i.e. additional training in research, education or in a clinical fellowship)?

- Yes
- No

4.a. Which additional training/degree(s) have you completed? (select all that apply)

- MSc (in research or education e.g. MBA, MA, MPH, MMEd etc.)
- PhD (in research or education)
- Post-doctoral fellowship (in research or education)
- Clinical fellowship (glomerulonephritis, dialysis, etc.)
- Other (please specify) ________________________

4.b. Why did you pursue this training/ degree? (select all that apply)

- I obtained it before beginning nephrology training (e.g. before or during medical school)
- I was directed by colleagues, including my division head/director; it was a pre-requisite for a job
- Although not directed to get my degree, I believed it would improve my chances of gaining employment
- No job was available at the time I completed training thus extra education deferred full employment until a job became available
- This was not a pre-requisite for a job. I wanted to advance my personal passion or growth.
- Other (please specify) ________________________

4.c. Do you utilize the knowledge gained from this higher training/degree within your usual scope of practice? (e.g. – if you have a Masters in Medical Education are you regularly involved in educational activities?)

- Yes
- No
- Unsure

Simple Skipping Information

- If 5. During regular working hours, are you working <... = Yes then Skip to Page 11
- If 5. During regular working hours, are you working <... = No, I am working (at least partially) as a Nephrologist then Skip to Page 12

For the following questions, a Nephrologist is considered a physician whose practice involves caring for patients with significant kidney diseases including those approaching, or with, ESRD including patients who may have received a transplant or are currently receiving dialysis.

5. During regular working hours, are you working exclusively as a non-Nephrologist (e.g. only working as a general internist or general pediatrician)?

- Yes
- No, I am working (at least partially) as a Nephrologist

5.a. Is working exclusively as a non-Nephrologist your preferred choice?

- ◯ Yes
- ◯ No

5.b. If it is not your choice, why are you not working as a Nephrologist (please check all that apply)?

- ☐ I am unable to get a job as a Nephrologist at my site of practice
- ☐ There are no dialysis facilities in my area
- ☐ Other (please specify): ________________________

6. What resources were most useful when finding a Nephrologist job?

Please rank you top three choices:

	1	2	3
CSN meetings	◯	◯	◯
CSN website	◯	◯	◯
CMAJ	◯	◯	◯
Krescent Program	◯	◯	◯
Other research program	◯	◯	◯
Program Director	◯	◯	◯
Division Head	◯	◯	◯
Colleagues in other centres	◯	◯	◯
Word of mouth	◯	◯	◯
Social media	◯	◯	◯
Other	◯	◯	◯

If you choose other please specify:

____

7. What If any, challenges have you found in finding employment as a Nephrologist? (select all that apply)

- ☐ No challenge finding nephrology employment.
- ☐ I could not find a nephrology job in Canada.
- ☐ I could not find a nephrology job in the location of my choice.
- ☐ I could not find a nephrology job where my particular skills (e.g. research expertise) could be used.
- ☐ Non-nephrology job related factors; e.g. No job available for my spouse or significant other.
- ☐ Job vacancies appeared to be filled/candidate selection made prior to the advertisement posting.
- ☐ Canadian immigration, language, or training restrictions/ requirments.
- ☐ Difficulty identifying job vacancies in Canada due to inconsistent posting/advertising practices.
- ☐ Other (please specify) ________________________

Simple Skipping Information

- If 9. Please choose the best answer that define your ... = I practice Adult nephrology only. then Skip to Page 15
- If 9. Please choose the best answer that define your ... = I practice Pediatric nephrology only. then Skip to Page 15
- If 9. Please choose the best answer that define your ... = I practice both Adult and Pediatric nephrology. then Skip to Page 15
- If 9. Please choose the best answer that define your ... = I practice Adult Nephrology and at least one other Another Specialty (e.g. GIM) then Skip to Page 14
- If 9. Please choose the best answer that define your ... = I practice Pediatric Nephrology and at least one other Pediatric Specialty (e.g.General Pediatrics). then Skip to Page 14

8. Including yourself, how many physicians practice Nephrology within your primary catchment area?

____

9. Please choose the best answer that define your practice

- ◯ I practice Adult nephrology only.
- ◯ I practice Pediatric nephrology only.
- ◯ I practice both Adult and Pediatric nephrology.
- ◯ I practice Adult Nephrology and at least one other Another Specialty (e.g. GIM)
- ◯ I practice Pediatric Nephrology and at least one other Pediatric Specialty (e.g.General Pediatrics).

9.a. My other specialty(s) that I practice is (select all that apply):

- [ ] General Internal Medicine
- [ ] General Pediatrics
- [ ] ICU
- [ ] Other (please specify): ________________________

10. Please choose the best answer(s) that define your practice

- ◯ My practice is solo.
- ◯ My practice is group.

If you are participating in a 'primary Nephrologist' model (where your patients can identify you as their Nephrologist), please answer question 11. If your patients would not routinely identify you as their primary Nephrologist please proceed to question 12.

11. Approximately how many of the following patients do you follow within your practice as a primary nephrologist exclusively;

Stage 1-3 CKD	____________________
Non dialysis CKD stage 3-5	____________________
ESRD on peritoneal dialysis	____________________
ESRD on hemodialysis	____________________
Post-transplant	____________________

12. Do you routinely 'share' the care of your outpatients with other Nephrologists (please check all that apply)?

- ☐ No, I am the primary Nephrologist for my outpatients
- ☐ Yes, we share outpatients with ESRD on dialysis
- ☐ Yes we share post-transplant outpatients
- ☐ Yes we share all outpatients CKD, ESRD, and transplant outpatients
- ☐ I am in a different model: (please specify) ________________________

13. Please choose the best answer(s) that define your practice

- ◯ My practice is Academic only.
- ◯ My practice is Community only.
- ◯ My practice is Community but affiliated with an Academic center.

14. What best describes your primary source(s) of physician income?

- ◯ I am in an Academic Alternative Relationship/Funding Plan (not fee for service) for 100% financial compensation.
- ◯ I am 100% Fee-for-Service (billings to Provincial Health Plan) compensation.
- ◯ I receive a combination of ARP/AFP and Fee-for-Service compensation for all my activities
- ◯ I am on salary - non Academic Alternative Relationship Funding Plan (e.g.: capitation payment system, WCB, government or hospital administration salary)
- ◯ Other (please describe): ________________________

15. Please describe the practice mix of your usual working year:

Type of activity	Percent of usual working year (total 100)
Clinical Nephrology, including direct inpatient/outpatient care for patients with kidney disease (EXCLUDING renal transplant patients)	____________________ __
Clinical Nephrology, renal transplant ONLY	____________________ __
General Internal Medicine	____________________ __
General Pediatrics	____________________ __
Intensive Care Unit Attending	____________________ __

Pediatric Intensive Care Unit Attending	____________________ __
Teaching (includes undergraduate, postgraduate and fellows)	____________________ __
Administration	____________________ __
Research	____________________ __
Other (i.e. CME, etc.)	____________________ __

16. Do you have access to nurse practitioners, physician assistants, clinical associates or advanced practice nurses to offset the usual daily work of a Nephrologist and to help provide regular care?

For this question we are not including charge nurses for dialysis units or hospital wards.

- [ ] Yes, these practitioners help care for my hemodialysis patients
- [ ] Yes, these practitioners help care for my peritoneal dialysis patients
- [ ] Yes, these practitioners help care for my outpatients with stage 3-5 CKD
- [ ] Yes, these practitioners help care for my transplant program patients
- [ ] Yes, these practitioners help care for my hospital inpatients.
- [ ] Yes, I have access to these practitioners who assist me but not in the above ways _________________________
- [ ] No, I do not have access to these types of practitioners to care for my nephrology patients

16.a. If you answered yes to any of the above options in question 16, what type(s) of assistant do you have

For this question we are not including charge nurses for dialysis units or hospital wards.

- [ ] Nurse practitioner
- [ ] Physician assistant
- [ ] Pharmacist
- [ ] Clinical associates
- [ ] Other (please specify) _________________________

17. Are you satisfied with your current position?

not satisfied at all — somewhat satisfied — satisfied — very satisfied

1	2	3	4	5	6	7	8	9	10
○	○	○	○	○	○	○	○	○	○

Simple Skipping Information

- If 5. Do you plan to decrease the number of daytime h... = 3 years then Skip to Page 17
- If 5. Do you plan to decrease the number of daytime h... = 5 years then Skip to Page 18
- If 5. Do you plan to decrease the number of daytime h... = 10 years then Skip to Page 19
- If 5. Do you plan to decrease the number of daytime h... = 15 years then Skip to Page 20
- If 5. Do you plan to decrease the number of daytime h... = Unsure then Skip to Page 21
- If 5. Do you plan to decrease the number of daytime h... = Not within 15 years then Skip to Page 21

WORK HOURS

1.On average, over the past year, how many hours per week do you work as a Nephrologist (exclude on call)

0-10 hours	11 – 35 hours	36 – 40 hours	41 – 50 hours	51 – 60 hours	61 – 80 hours	81 – 100 hours	>100 hours
◯	◯	◯	◯	◯	◯	◯	◯

2. Has the number of daytime hours per week that you work:

◯ Increased in the past 2 years
◯ Decreased in the past 2 years
◯ Not changed in the past 2 years

3. During the past year, including weekends, approximately how many nights were you on call as a nephrologist?

____

4. Has the number of on-call hours per week that you work:

◯ Increased in the past 2 years
◯ Decreased in the past 2 years
◯ Not changed in the past 2 years

5. Do you plan to decrease the number of daytime hours you work in the next:

◯ 3 years
◯ 5 years
◯ 10 years
◯ 15 years
◯ Unsure
◯ Not within 15 years

In the next 3 years you plan on decreasing your number of daytime hours by what percent?

____

In the next 5 years you plan on decreasing your number of daytime hours by what percent?

____

In the next 10 years you plan on decreasing your number of daytime hours by what percent?

____

In the next 15 years you plan on decreasing your number of daytime hours by what <u>percent</u>?

____

6. Are you planning to stop working (i.e. completely retire) in the next:

- 3 years
- 5 years
- 10 years
- 15 years
- Unsure
- I do not plan on retiring in the next 15 years

7. Are you satisfied with the work hours in your current position?

not satisfied at all / somewhat satisfied / satisfied / very satisfied

1	2	3	4	5	6	7	8	9	10
○	○	○	○	○	○	○	○	○	○

Simple Skipping Information

- If Are you the division head or, lead responsible for... = Yes then Skip to Page 23
- If Are you the division head or, lead responsible for... = No then Skip to Page 24

WE ARE SEEKING ADDITIONAL INFORMATION FROM NEPHROLOGISTS WHO HAVE THE AUTHORITY OR INFLUENCE IN HIRING NEPHROLOGISTS IN YOUR GEOGRAPHIC AREA

Are you the division head or, lead responsible for recruitment for nephrology in your area?

- Yes
- No

1. How many clinical Nephrologists are in your program? (defined as >75% of time spent on patient care related activities)

____

2. How many academic Nephrologists are in your program? (defined as <75% of time spent on patient care related activities, with the remainder spent on research, teaching or administration)

____

For the following, a FTE is defined as 45-50h per week, for 44 weeks per year (2200h/year) of employment.

3. Of the clinical (defined as >75% of time spent on patient care related activities) Nephrologists in your program, how many are employed as:

1.0 FTE	____________________
0.75 FTE	____________________
0.50 FTE	____________________
0.25 FTE	____________________

4. Of the academic (defined as <75% of time spent on patient care related activities) Nephrologists in your program, how many are employed as:

1.0 FTE	____________________
0.75 FTE	____________________
0.50 FTE	____________________
0.25 FTE	____________________

5. What determines recruitment of clinical Nephrologists in your centre (please select all that apply)?

- ☐ Increased patient numbers
- ☐ Members decreasing workload
- ☐ Mandated or limited by Renal Program administration
- ☐ Increased patient complexity
- ☐ Other (please specify) ________________________

6. Assuming that funding and resources were assured and the administration was supportive, how many additional FTE clinical and academic Nephrologists does your program need?

Number of additional clinical Nephrolgists needed:

Now	____________________
Within 3 years	____________________
Within 5 years	____________________

Number of additional academic Nephrologists:

Now	____________________
Within 3 years	____________________
Within 5 years	____________________

7. What determines recruitment of academic Nephrologists in your center (please select all that apply)?

- ☐ Increased teaching requirements

- Increased patient numbers
- Members decreasing workload
- Mandated from renal program administration
- University priority or mandate
- Other (please specify) ________________________

8. If you are seeking to recruit a academic Nephrologist, but are unable to, why (please select all that apply)?

- Availability of positions
- Unable to find suitable candidate
- Lack of funding
- Will of the division
- Funding for the academic portion of the salary
- Other (please specify) ________________________

9. If you are seeking to recruit a clinical Nephrologist, but are unable to, why (please select all that apply)?

- Availability of positions
- Unable to find suitable candidate
- Lack of funding
- Will of the division
- Other (please specify) ________________________

10. Are there other issues about nephrology recruitment or workforce planning you would like to share?

____

IF YOU WOULD LIKE TO BE ENTERED INTO A DRAW FOR AN APPLE IPAD PLEASE ENTER YOUR EMAIL ADDRESS BELOW. THE WINNER WILL BE CONTACTED WITHIN 48H OF THE CLOSING OF THE SURVEY.

____

THANK YOU FOR TAKING THE TIME TO FILL OUT THIS SURVEY
